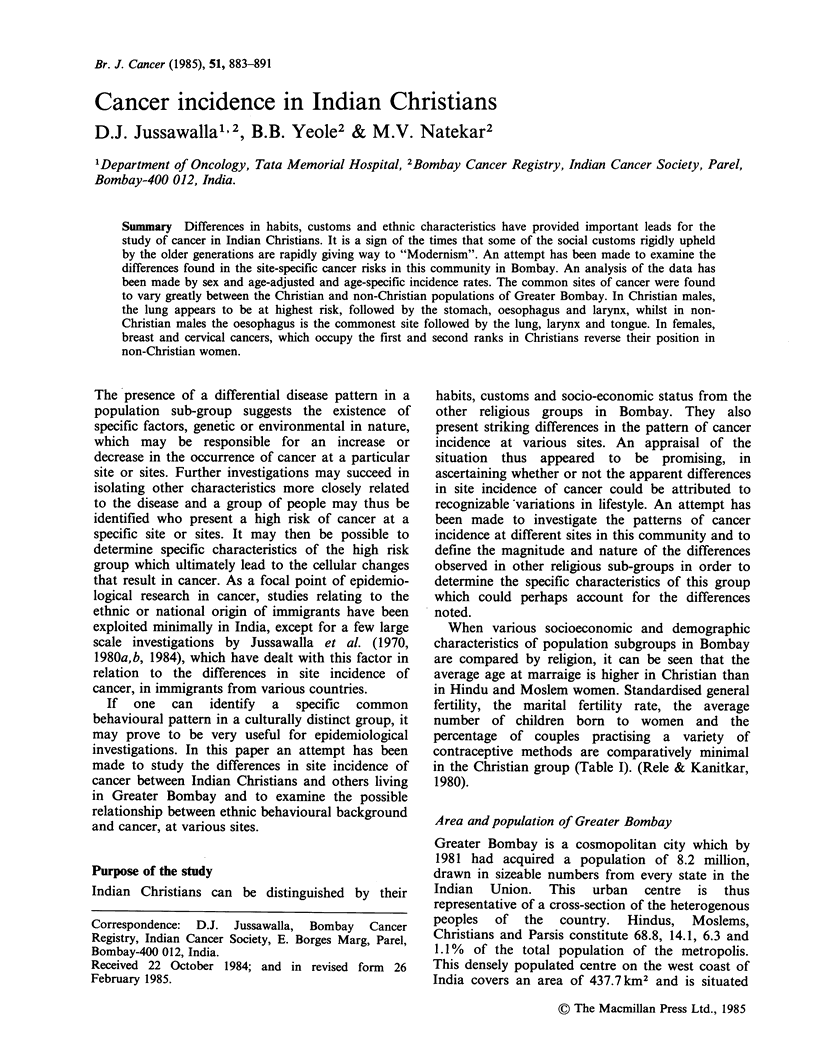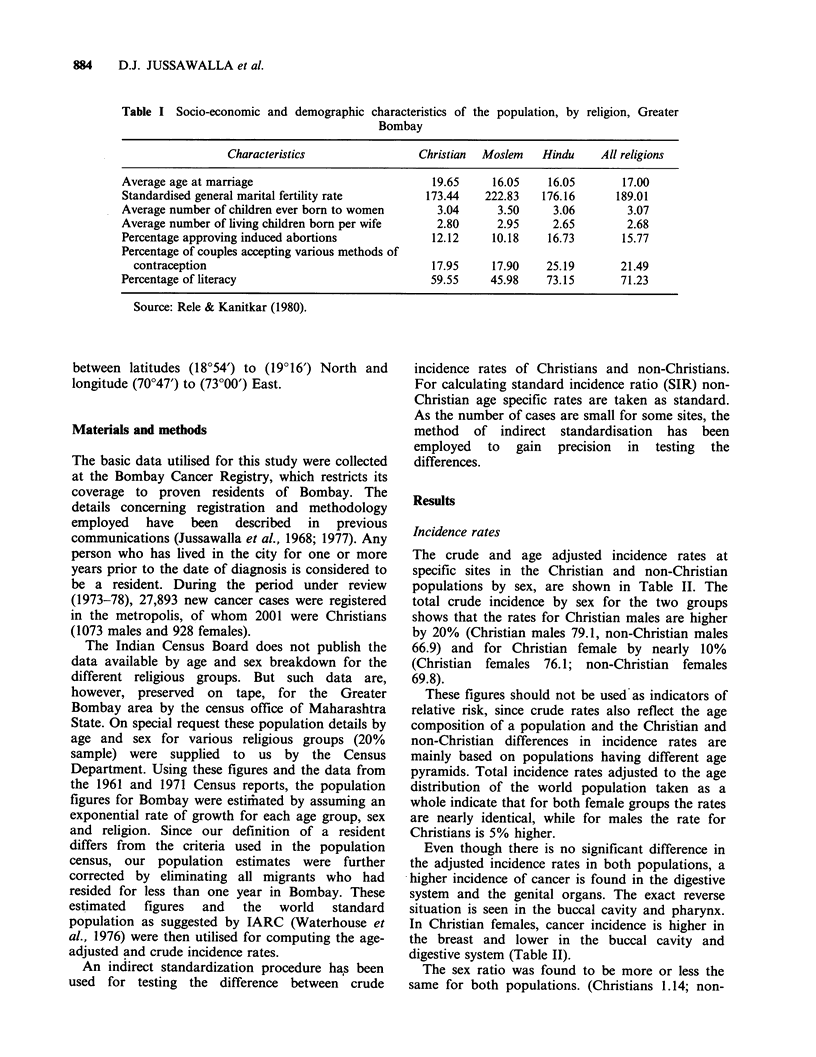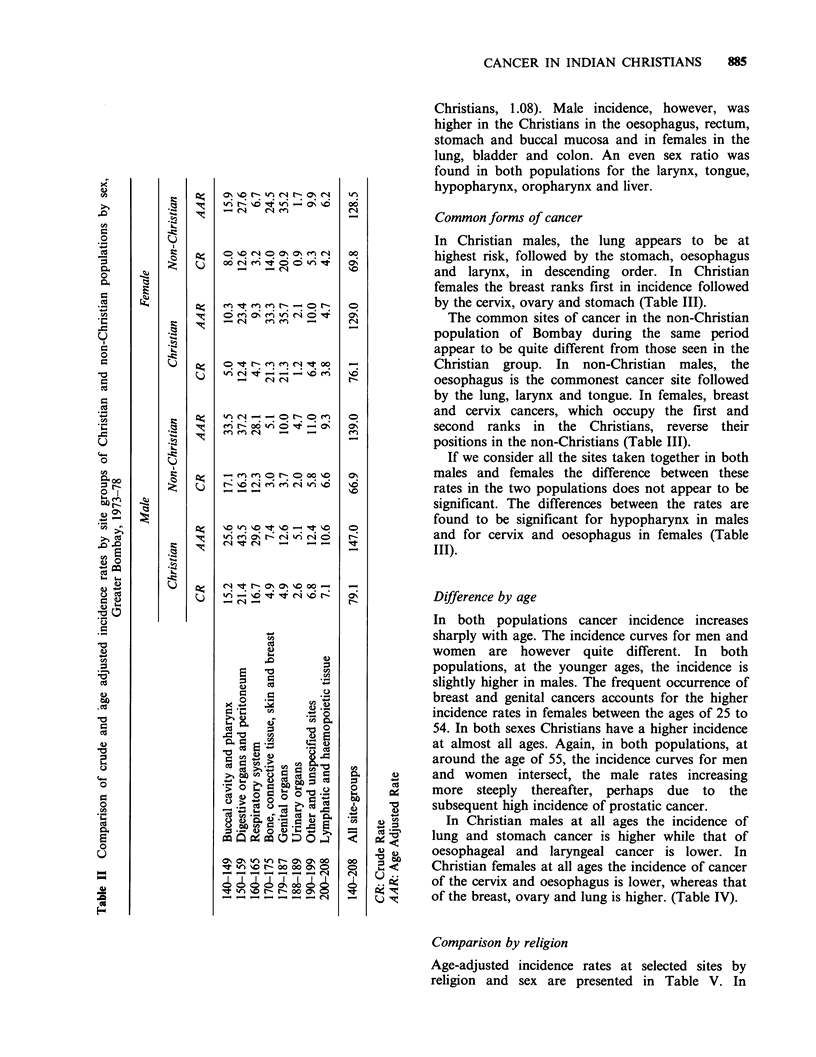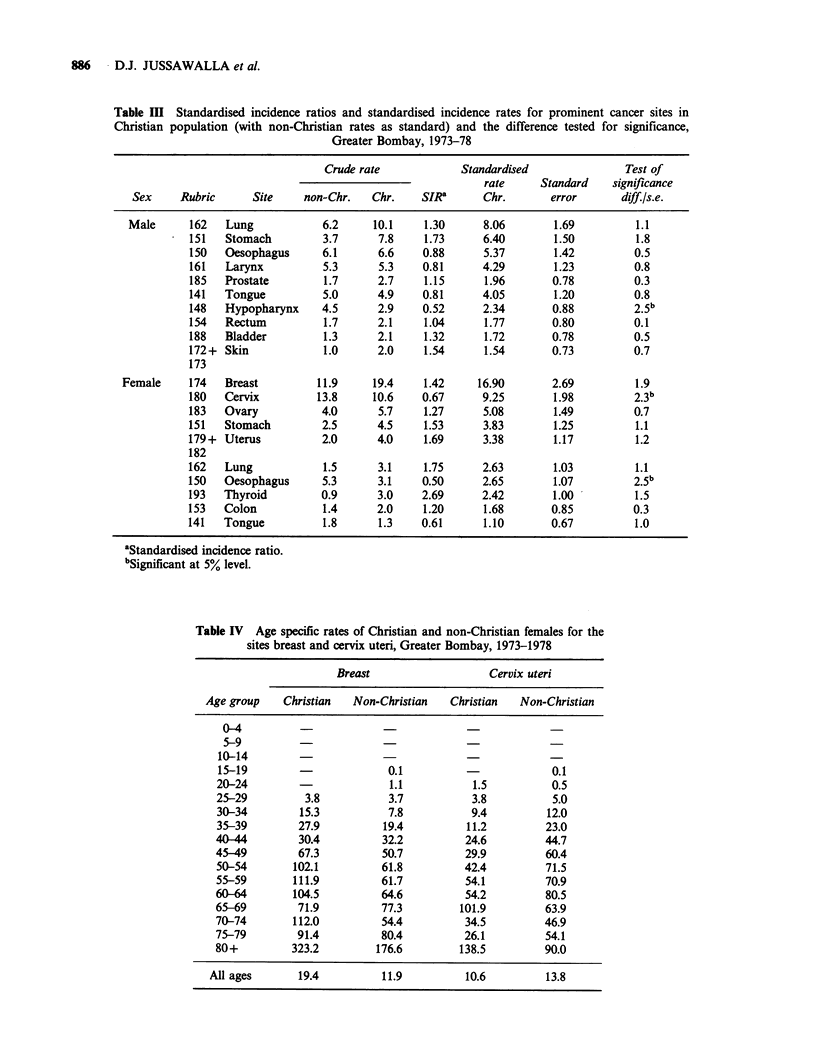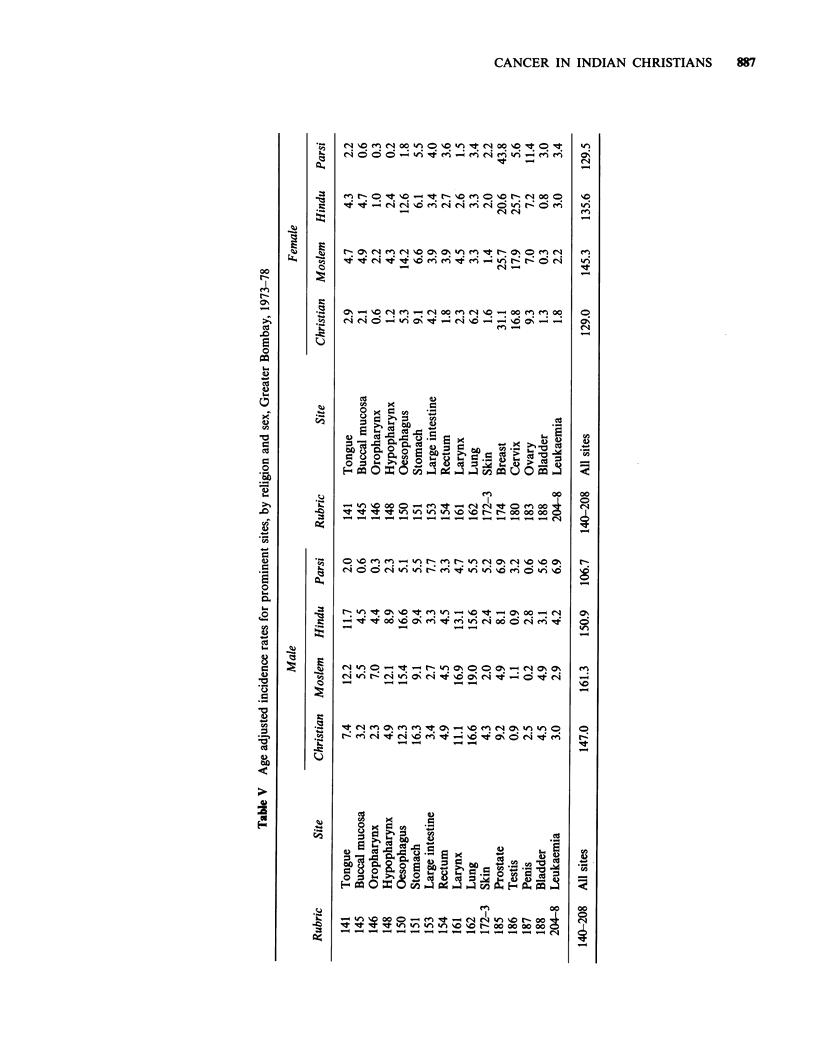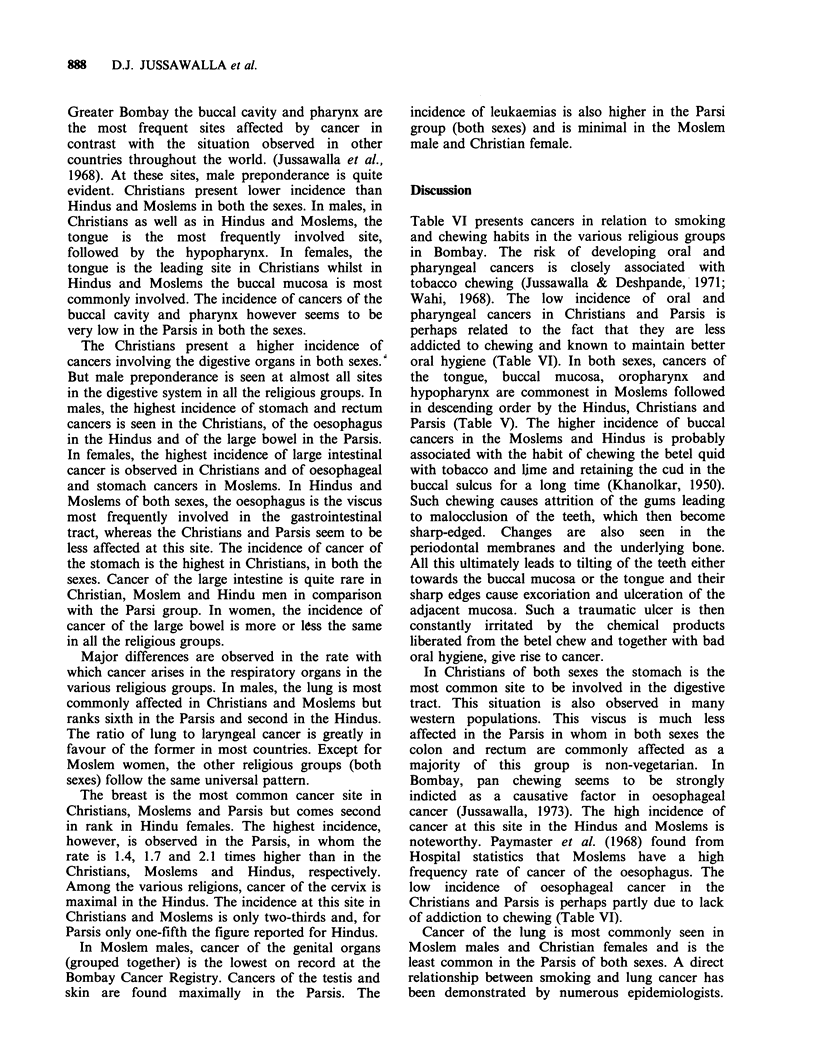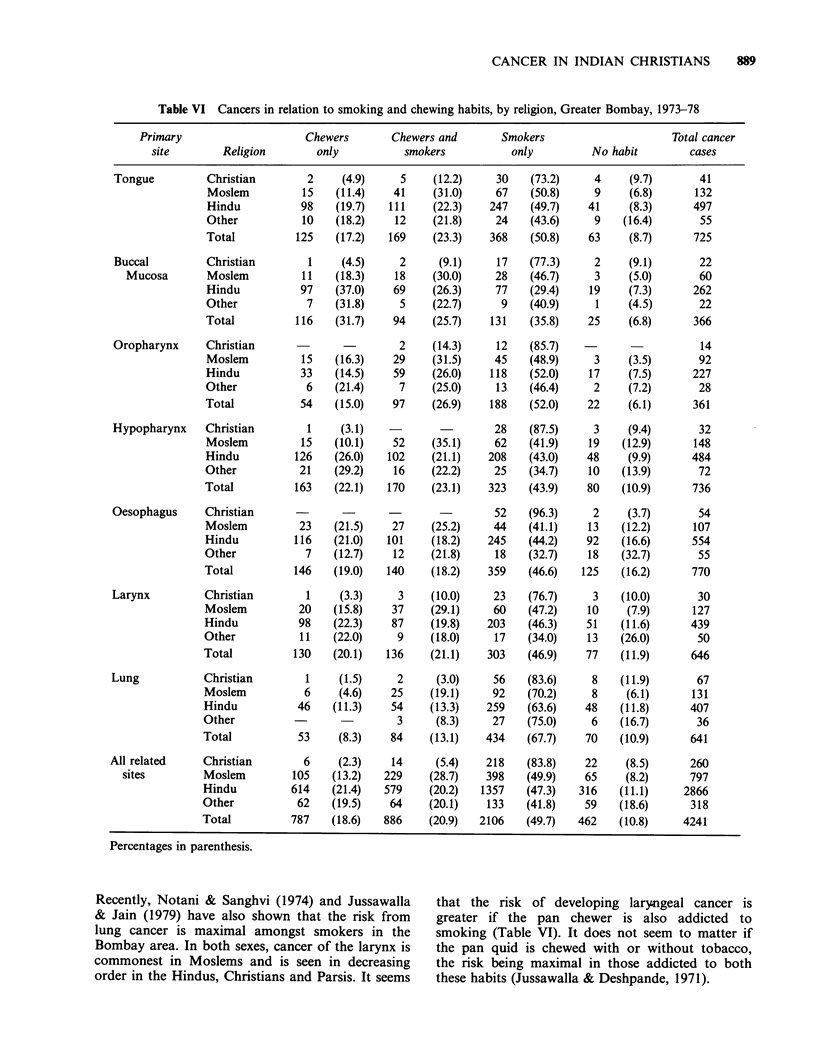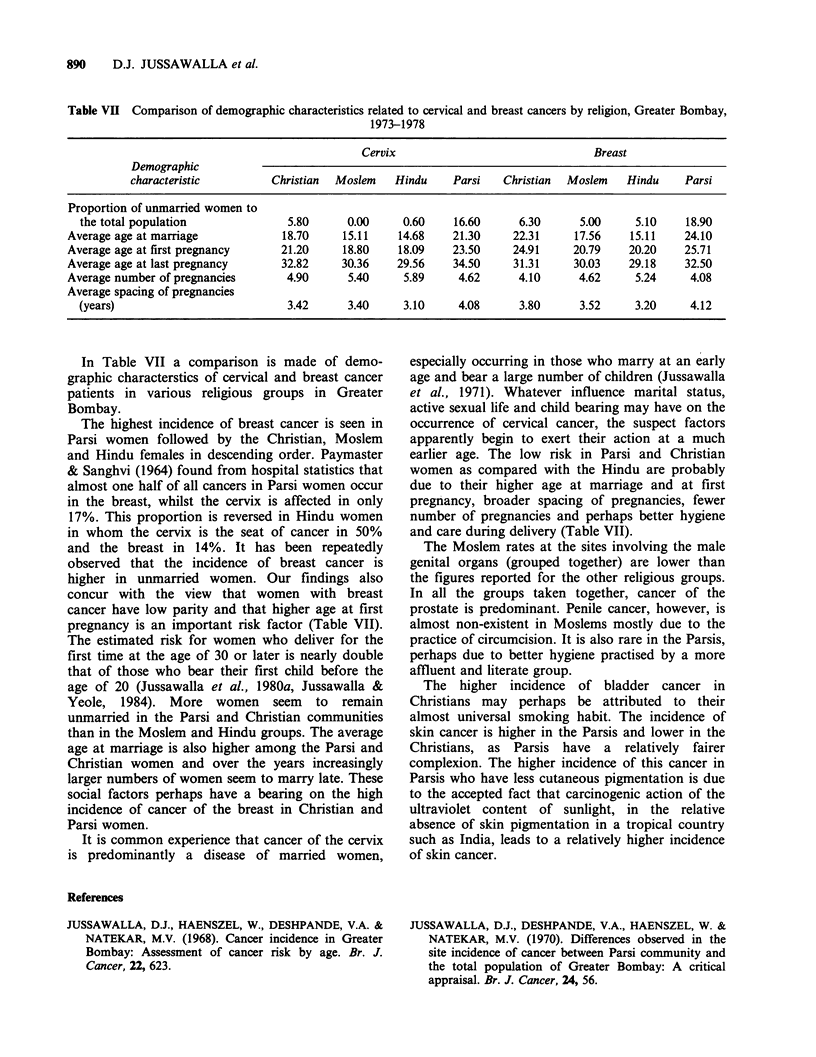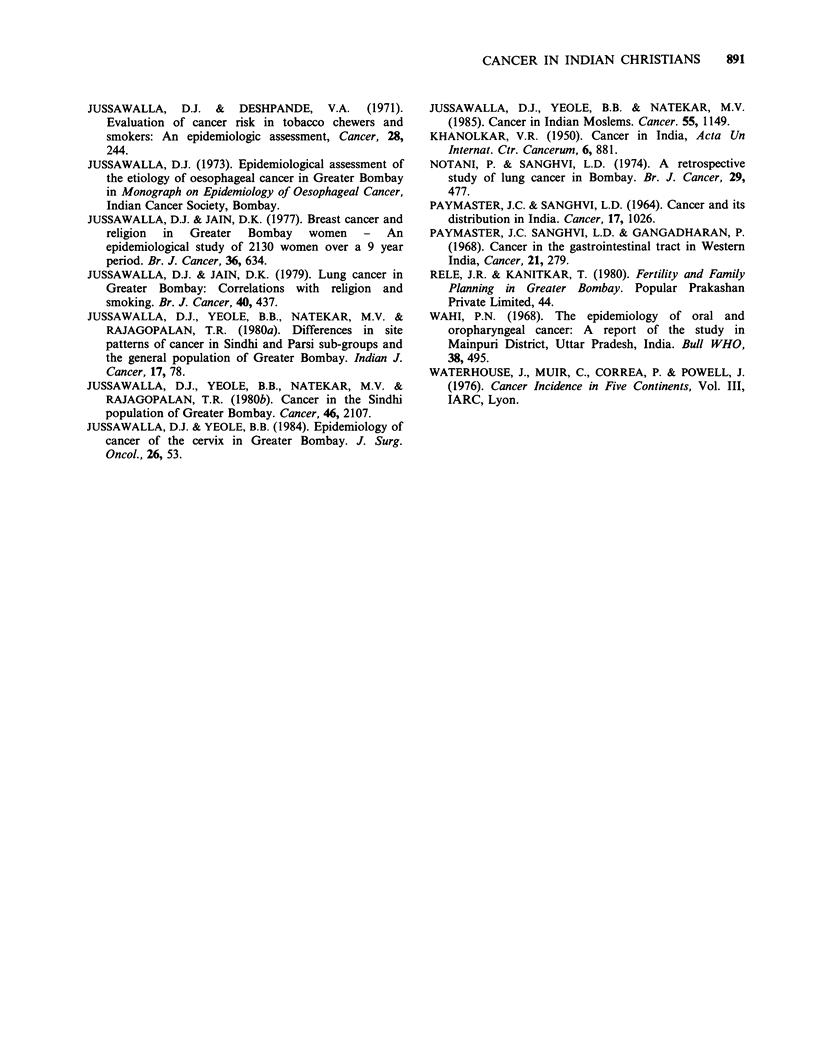# Cancer incidence in Indian Christians.

**DOI:** 10.1038/bjc.1985.134

**Published:** 1985-06

**Authors:** D. J. Jussawalla, B. B. Yeole, M. V. Natekar

## Abstract

Differences in habits, customs and ethnic characteristics have provided important leads for the study of cancer in Indian Christians. It is a sign of the times that some of the social customs rigidly upheld by the older generations are rapidly giving way to "Modernism". An attempt has been made to examine the differences found in the site-specific cancer risks in this community in Bombay. An analysis of the data has been made by sex and age-adjusted and age-specific incidence rates. The common sites of cancer were found to vary greatly between the Christian and non-Christian populations of Greater Bombay. In Christian males, the lung appears to be at highest risk, followed by the stomach, oesophagus and larynx, whilst in non-Christian males the oesophagus is the commonest site followed by the lung, larynx and tongue. In females, breast and cervical cancers, which occupy the first and second ranks in Christians reverse their position in non-Christian women.


*Br. J. Cancer* (1985), **51**, 883–891

### Cancer incidence in Indian Christians

D.J. Jussawalla[1,2], B.B. Yeole[2] & M.V. Natekar[2]

[1]*Department of Oncology, Tata Memorial Hospital,* [2]*Bombay Cancer Registry, Indian Cancer Society, Parel, Bombay-400 012, India.*

**Summary** Differences in habits, customs and ethnic characteristics have provided important leads for the study of cancer in Indian Christians. It is a sign of the times that some of the social customs rigidly upheld by the older generations are rapidly giving way to "Modernism". An attempt has been made to examine the differences found in the site-specific cancer risks in this community in Bombay. An analysis of the data has been made by sex and age-adjusted and age-specific incidence rates. The common sites of cancer were found to vary greatly between the Christian and non-Christian populations of Greater Bombay. In Christian males, the lung appears to be at highest risk, followed by the stomach, oesophagus and larynx, whilst in non-Christian males the oesophagus is the commonest site followed by the lung, larynx and tongue. In females, breast and cervical cancers, which occupy the first and second ranks in Christians reverse their position in non-Christian women.

The presence of a differential disease pattern in a population sub-group suggests the existence of specific factors, genetic or environmental in nature, which may be responsible for an increase or decrease in the occurrence of cancer at a particular site or sites. Further investigations may succeed in isolating other characteristics more closely related to the disease and a group of people may thus be identified who present a high risk of cancer at a specific site or sites. It may then be possible to determine specific characteristics of the high risk group which ultimately lead to the cellular changes that result in cancer. As a focal point of epidemiological research in cancer, studies relating to the ethnic or national origin of immigrants have been exploited minimally in India, except for a few large scale investigations by Jussawalla *et al.* (1970, 1980*a,b*, 1984), which have dealt with this factor in relation to the differences in site incidence of cancer, in immigrants from various countries.

If one can identify a specific common behavioural pattern in a culturally distinct group, it may prove to be very useful for epidemiological investigations. In this paper an attempt has been made to study the differences in site incidence of cancer between Indian Christians and others living in Greater Bombay and to examine the possible relationship between ethnic behavioural background and cancer, at various sites.

### Purpose of the study

Indian Christians can be distinguished by their habits, customs and socio-economic status from the other religious groups in Bombay. They also present striking differences in the pattern of cancer incidence at various sites. An appraisal of the situation thus appeared to be promising, in ascertaining whether or not the apparent differences in site incidence of cancer could be attributed to recognizable variations in lifestyle. An attempt has been made to investigate the patterns of cancer incidence at different sites in this community and to define the magnitude and nature of the differences observed in other religious sub-groups in order to determine the specific characteristics of this group which could perhaps account for the differences noted.

When various socioeconomic and demographic characteristics of population subgroups in Bombay are compared by religion, it can be seen that the average age at marraige is higher in Christian than in Hindu and Moslem women. Standardised general fertility, the marital fertility rate, the average number of children born to women and the percentage of couples practising a variety of contraceptive methods are comparatively minimal in the Christian group (Table I). (Rele & Kanitkar, 1980).

### *Area and population of Greater Bombay*

Greater Bombay is a cosmopolitan city which by 1981 had acquired a population of 8.2 million, drawn in sizeable numbers from every state in the Indian Union. This urban centre is thus representative of a cross-section of the heterogenous peoples of the country. Hindus, Moslems, Christians and Parsis constitute 68.8, 14.1, 6.3 and 1.1% of the total population of the metropolis. This densely populated centre on the west coast of India covers an area of 437.7 km$^2$ and is situated

Correspondence: D.J. Jussawalla, Bombay Cancer Registry, Indian Cancer Society, E. Borges Marg, Parel, Bombay-400 012, India.
Received 22 October 1984; and in revised form 26 February 1985.

© The Macmillan Press Ltd., 1985

**Table I** Socio-economic and demographic characteristics of the population, by religion, Greater Bombay

| *Characteristics* | *Christian* | *Moslem* | *Hindu* | *All religions* |
|---|---|---|---|---|
| Average age at marriage | 19.65 | 16.05 | 16.05 | 17.00 |
| Standardised general marital fertility rate | 173.44 | 222.83 | 176.16 | 189.01 |
| Average number of children ever born to women | 3.04 | 3.50 | 3.06 | 3.07 |
| Average number of living children born per wife | 2.80 | 2.95 | 2.65 | 2.68 |
| Percentage approving induced abortions | 12.12 | 10.18 | 16.73 | 15.77 |
| Percentage of couples accepting various methods of contraception | 17.95 | 17.90 | 25.19 | 21.49 |
| Percentage of literacy | 59.55 | 45.98 | 73.15 | 71.23 |

Source: Rele & Kanitkar (1980).

between latitudes (18°54′) to (19°16′) North and longitude (70°47′) to (73°00′) East.

### Materials and methods

The basic data utilised for this study were collected at the Bombay Cancer Registry, which restricts its coverage to proven residents of Bombay. The details concerning registration and methodology employed have been described in previous communications (Jussawalla *et al.*, 1968; 1977). Any person who has lived in the city for one or more years prior to the date of diagnosis is considered to be a resident. During the period under review (1973–78), 27,893 new cancer cases were registered in the metropolis, of whom 2001 were Christians (1073 males and 928 females).

The Indian Census Board does not publish the data available by age and sex breakdown for the different religious groups. But such data are, however, preserved on tape, for the Greater Bombay area by the census office of Maharashtra State. On special request these population details by age and sex for various religious groups (20% sample) were supplied to us by the Census Department. Using these figures and the data from the 1961 and 1971 Census reports, the population figures for Bombay were estimated by assuming an exponential rate of growth for each age group, sex and religion. Since our definition of a resident differs from the criteria used in the population census, our population estimates were further corrected by eliminating all migrants who had resided for less than one year in Bombay. These estimated figures and the world standard population as suggested by IARC (Waterhouse *et al.*, 1976) were then utilised for computing the age-adjusted and crude incidence rates.

An indirect standardization procedure has been used for testing the difference between crude incidence rates of Christians and non-Christians. For calculating standard incidence ratio (SIR) non-Christian age specific rates are taken as standard. As the number of cases are small for some sites, the method of indirect standardisation has been employed to gain precision in testing the differences.

### Results

### *Incidence rates*

The crude and age adjusted incidence rates at specific sites in the Christian and non-Christian populations by sex, are shown in Table II. The total crude incidence by sex for the two groups shows that the rates for Christian males are higher by 20% (Christian males 79.1, non-Christian males 66.9) and for Christian female by nearly 10% (Christian females 76.1; non-Christian females 69.8).

These figures should not be used as indicators of relative risk, since crude rates also reflect the age composition of a population and the Christian and non-Christian differences in incidence rates are mainly based on populations having different age pyramids. Total incidence rates adjusted to the age distribution of the world population taken as a whole indicate that for both female groups the rates are nearly identical, while for males the rate for Christians is 5% higher.

Even though there is no significant difference in the adjusted incidence rates in both populations, a higher incidence of cancer is found in the digestive system and the genital organs. The exact reverse situation is seen in the buccal cavity and pharynx. In Christian females, cancer incidence is higher in the breast and lower in the buccal cavity and digestive system (Table II).

The sex ratio was found to be more or less the same for both populations. (Christians 1.14; non-

**Table II** Comparison of crude and age adjusted incidence rates by site groups of Christian and non-Christian populations by sex, Greater Bombay, 1973–78

| | | Male | | | | Female | | | |
|---|---|---|---|---|---|---|---|---|---|
| | | *Christian* | | *Non-Christian* | | *Christian* | | *Non-Christian* | |
| | | *CR* | *AAR* | *CR* | *AAR* | *CR* | *AAR* | *CR* | *AAR* |
| 140–149 | Buccal cavity and pharynx | 15.2 | 25.6 | 17.1 | 33.5 | 5.0 | 10.3 | 8.0 | 15.9 |
| 150–159 | Digestive organs and peritoneum | 21.4 | 43.5 | 16.3 | 37.2 | 12.4 | 23.4 | 12.6 | 27.6 |
| 160–165 | Respiratory system | 16.7 | 29.6 | 12.3 | 28.1 | 4.7 | 9.3 | 3.2 | 6.7 |
| 170–175 | Bone, connective tissue, skin and breast | 4.9 | 7.4 | 3.0 | 5.1 | 21.3 | 33.3 | 14.0 | 24.5 |
| 179–187 | Genital organs | 4.9 | 12.6 | 3.7 | 10.0 | 21.3 | 35.7 | 20.9 | 35.2 |
| 188–189 | Urinary organs | 2.6 | 5.1 | 2.0 | 4.7 | 1.2 | 2.1 | 0.9 | 1.7 |
| 190–199 | Other and unspecified sites | 6.8 | 12.4 | 5.8 | 11.0 | 6.4 | 10.0 | 5.3 | 9.9 |
| 200–208 | Lymphatic and haemopoietic tissue | 7.1 | 10.6 | 6.6 | 9.3 | 3.8 | 4.7 | 4.2 | 6.2 |
| 140–208 | All site-groups | 79.1 | 147.0 | 66.9 | 139.0 | 76.1 | 129.0 | 69.8 | 128.5 |

*CR*: Crude Rate

*AAR*: Age Adjusted Rate

Christians, 1.08). Male incidence, however, was higher in the Christians in the oesophagus, rectum, stomach and buccal mucosa and in females in the lung, bladder and colon. An even sex ratio was found in both populations for the larynx, tongue, hypopharynx, oropharynx and liver.

*Common forms of cancer*

In Christian males, the lung appears to be at highest risk, followed by the stomach, oesophagus and larynx, in descending order. In Christian females the breast ranks first in incidence followed by the cervix, ovary and stomach (Table III).

The common sites of cancer in the non-Christian population of Bombay during the same period appear to be quite different from those seen in the Christian group. In non-Christian males, the oesophagus is the commonest cancer site followed by the lung, larynx and tongue. In females, breast and cervix cancers, which occupy the first and second ranks in the Christians, reverse their positions in the non-Christians (Table III).

If we consider all the sites taken together in both males and females the difference between these rates in the two populations does not appear to be significant. The differences between the rates are found to be significant for hypopharynx in males and for cervix and oesophagus in females (Table III).

*Difference by age*

In both populations cancer incidence increases sharply with age. The incidence curves for men and women are however quite different. In both populations, at the younger ages, the incidence is slightly higher in males. The frequent occurrence of breast and genital cancers accounts for the higher incidence rates in females between the ages of 25 to 54. In both sexes Christians have a higher incidence at almost all ages. Again, in both populations, at around the age of 55, the incidence curves for men and women intersect, the male rates increasing more steeply thereafter, perhaps due to the subsequent high incidence of prostatic cancer.

In Christian males at all ages the incidence of lung and stomach cancer is higher while that of oesophageal and laryngeal cancer is lower. In Christian females at all ages the incidence of cancer of the cervix and oesophagus is lower, whereas that of the breast, ovary and lung is higher. (Table IV).

*Comparison by religion*

Age-adjusted incidence rates at selected sites by religion and sex are presented in Table V. In

**Table III** Standardised incidence ratios and standardised incidence rates for prominent cancer sites in Christian population (with non-Christian rates as standard) and the difference tested for significance, Greater Bombay, 1973–78

| Sex | Rubric | Site | Crude rate non-Chr. | Crude rate Chr. | SIR[a] | Standardised rate Chr. | Standard error | Test of significance diff./s.e. |
|---|---|---|---|---|---|---|---|---|
| Male | 162 | Lung | 6.2 | 10.1 | 1.30 | 8.06 | 1.69 | 1.1 |
| | 151 | Stomach | 3.7 | 7.8 | 1.73 | 6.40 | 1.50 | 1.8 |
| | 150 | Oesophagus | 6.1 | 6.6 | 0.88 | 5.37 | 1.42 | 0.5 |
| | 161 | Larynx | 5.3 | 5.3 | 0.81 | 4.29 | 1.23 | 0.8 |
| | 185 | Prostate | 1.7 | 2.7 | 1.15 | 1.96 | 0.78 | 0.3 |
| | 141 | Tongue | 5.0 | 4.9 | 0.81 | 4.05 | 1.20 | 0.8 |
| | 148 | Hypopharynx | 4.5 | 2.9 | 0.52 | 2.34 | 0.88 | 2.5[b] |
| | 154 | Rectum | 1.7 | 2.1 | 1.04 | 1.77 | 0.80 | 0.1 |
| | 188 | Bladder | 1.3 | 2.1 | 1.32 | 1.72 | 0.78 | 0.5 |
| | 172+ 173 | Skin | 1.0 | 2.0 | 1.54 | 1.54 | 0.73 | 0.7 |
| Female | 174 | Breast | 11.9 | 19.4 | 1.42 | 16.90 | 2.69 | 1.9 |
| | 180 | Cervix | 13.8 | 10.6 | 0.67 | 9.25 | 1.98 | 2.3[b] |
| | 183 | Ovary | 4.0 | 5.7 | 1.27 | 5.08 | 1.49 | 0.7 |
| | 151 | Stomach | 2.5 | 4.5 | 1.53 | 3.83 | 1.25 | 1.1 |
| | 179+ 182 | Uterus | 2.0 | 4.0 | 1.69 | 3.38 | 1.17 | 1.2 |
| | 162 | Lung | 1.5 | 3.1 | 1.75 | 2.63 | 1.03 | 1.1 |
| | 150 | Oesophagus | 5.3 | 3.1 | 0.50 | 2.65 | 1.07 | 2.5[b] |
| | 193 | Thyroid | 0.9 | 3.0 | 2.69 | 2.42 | 1.00 | 1.5 |
| | 153 | Colon | 1.4 | 2.0 | 1.20 | 1.68 | 0.85 | 0.3 |
| | 141 | Tongue | 1.8 | 1.3 | 0.61 | 1.10 | 0.67 | 1.0 |

[a]Standardised incidence ratio.
[b]Significant at 5% level.

**Table IV** Age specific rates of Christian and non-Christian females for the sites breast and cervix uteri, Greater Bombay, 1973–1978

| Age group | Breast Christian | Breast Non-Christian | Cervix uteri Christian | Cervix uteri Non-Christian |
|---|---|---|---|---|
| 0–4 | — | — | — | — |
| 5–9 | — | — | — | — |
| 10–14 | — | — | — | — |
| 15–19 | — | 0.1 | — | 0.1 |
| 20–24 | — | 1.1 | 1.5 | 0.5 |
| 25–29 | 3.8 | 3.7 | 3.8 | 5.0 |
| 30–34 | 15.3 | 7.8 | 9.4 | 12.0 |
| 35–39 | 27.9 | 19.4 | 11.2 | 23.0 |
| 40–44 | 30.4 | 32.2 | 24.6 | 44.7 |
| 45–49 | 67.3 | 50.7 | 29.9 | 60.4 |
| 50–54 | 102.1 | 61.8 | 42.4 | 71.5 |
| 55–59 | 111.9 | 61.7 | 54.1 | 70.9 |
| 60–64 | 104.5 | 64.6 | 54.2 | 80.5 |
| 65–69 | 71.9 | 77.3 | 101.9 | 63.9 |
| 70–74 | 112.0 | 54.4 | 34.5 | 46.9 |
| 75–79 | 91.4 | 80.4 | 26.1 | 54.1 |
| 80+ | 323.2 | 176.6 | 138.5 | 90.0 |
| All ages | 19.4 | 11.9 | 10.6 | 13.8 |

**Table V** Age adjusted incidence rates for prominent sites, by religion and sex, Greater Bombay, 1973–78

| | | Male | | | | | | Female | | | |
|---|---|---|---|---|---|---|---|---|---|---|---|
| *Rubric* | *Site* | *Christian* | *Moslem* | *Hindu* | *Parsi* | *Rubric* | *Site* | *Christian* | *Moslem* | *Hindu* | *Parsi* |
| 141 | Tongue | 7.4 | 12.2 | 11.7 | 2.0 | 141 | Tongue | 2.9 | 4.7 | 4.3 | 2.2 |
| 145 | Buccal mucosa | 3.2 | 5.5 | 4.5 | 0.6 | 145 | Buccal mucosa | 2.1 | 4.9 | 4.7 | 0.6 |
| 146 | Oropharynx | 2.3 | 7.0 | 4.4 | 0.3 | 146 | Oropharynx | 0.6 | 2.2 | 1.0 | 0.3 |
| 148 | Hypopharynx | 4.9 | 12.1 | 8.9 | 2.3 | 148 | Hypopharynx | 1.2 | 4.3 | 2.4 | 0.2 |
| 150 | Oesophagus | 12.3 | 15.4 | 16.6 | 5.1 | 150 | Oesophagus | 5.3 | 14.2 | 12.6 | 1.8 |
| 151 | Stomach | 16.3 | 9.1 | 9.4 | 5.5 | 151 | Stomach | 9.1 | 6.6 | 6.1 | 5.5 |
| 153 | Large intestine | 3.4 | 2.7 | 3.3 | 7.7 | 153 | Large intestine | 4.2 | 3.9 | 3.4 | 4.0 |
| 154 | Rectum | 4.9 | 4.5 | 4.5 | 3.3 | 154 | Rectum | 1.8 | 3.9 | 2.7 | 3.6 |
| 161 | Larynx | 11.1 | 16.9 | 13.1 | 4.7 | 161 | Larynx | 2.3 | 4.5 | 2.6 | 1.5 |
| 162 | Lung | 16.6 | 19.0 | 15.6 | 5.5 | 162 | Lung | 6.2 | 3.3 | 3.3 | 3.4 |
| 172–3 | Skin | 4.3 | 2.0 | 2.4 | 5.2 | 172–3 | Skin | 1.6 | 1.4 | 2.0 | 2.2 |
| 185 | Prostate | 9.2 | 4.9 | 8.1 | 6.9 | 174 | Breast | 31.1 | 25.7 | 20.6 | 43.8 |
| 186 | Testis | 0.9 | 1.1 | 0.9 | 3.2 | 180 | Cervix | 16.8 | 17.9 | 25.7 | 5.6 |
| 187 | Penis | 2.5 | 0.2 | 2.8 | 0.6 | 183 | Ovary | 9.3 | 7.0 | 7.2 | 11.4 |
| 188 | Bladder | 4.5 | 4.9 | 3.1 | 5.6 | 188 | Bladder | 1.3 | 0.3 | 0.8 | 3.0 |
| 204–8 | Leukaemia | 3.0 | 2.9 | 4.2 | 6.9 | 204–8 | Leukaemia | 1.8 | 2.2 | 3.0 | 3.4 |
| 140–208 | All sites | 147.0 | 161.3 | 150.9 | 106.7 | 140–208 | All sites | 129.0 | 145.3 | 135.6 | 129.5 |

Greater Bombay the buccal cavity and pharynx are the most frequent sites affected by cancer in contrast with the situation observed in other countries throughout the world. (Jussawalla *et al.*, 1968). At these sites, male preponderance is quite evident. Christians present lower incidence than Hindus and Moslems in both the sexes. In males, in Christians as well as in Hindus and Moslems, the tongue is the most frequently involved site, followed by the hypopharynx. In females, the tongue is the leading site in Christians whilst in Hindus and Moslems the buccal mucosa is most commonly involved. The incidence of cancers of the buccal cavity and pharynx however seems to be very low in the Parsis in both the sexes.

The Christians present a higher incidence of cancers involving the digestive organs in both sexes. But male preponderance is seen at almost all sites in the digestive system in all the religious groups. In males, the highest incidence of stomach and rectum cancers is seen in the Christians, of the oesophagus in the Hindus and of the large bowel in the Parsis. In females, the highest incidence of large intestinal cancer is observed in Christians and of oesophageal and stomach cancers in Moslems. In Hindus and Moslems of both sexes, the oesophagus is the viscus most frequently involved in the gastrointestinal tract, whereas the Christians and Parsis seem to be less affected at this site. The incidence of cancer of the stomach is the highest in Christians, in both the sexes. Cancer of the large intestine is quite rare in Christian, Moslem and Hindu men in comparison with the Parsi group. In women, the incidence of cancer of the large bowel is more or less the same in all the religious groups.

Major differences are observed in the rate with which cancer arises in the respiratory organs in the various religious groups. In males, the lung is most commonly affected in Christians and Moslems but ranks sixth in the Parsis and second in the Hindus. The ratio of lung to laryngeal cancer is greatly in favour of the former in most countries. Except for Moslem women, the other religious groups (both sexes) follow the same universal pattern.

The breast is the most common cancer site in Christians, Moslems and Parsis but comes second in rank in Hindu females. The highest incidence, however, is observed in the Parsis, in whom the rate is 1.4, 1.7 and 2.1 times higher than in the Christians, Moslems and Hindus, respectively. Among the various religions, cancer of the cervix is maximal in the Hindus. The incidence at this site in Christians and Moslems is only two-thirds and, for Parsis only one-fifth the figure reported for Hindus.

In Moslem males, cancer of the genital organs (grouped together) is the lowest on record at the Bombay Cancer Registry. Cancers of the testis and skin are found maximally in the Parsis. The incidence of leukaemias is also higher in the Parsi group (both sexes) and is minimal in the Moslem male and Christian female.

### Discussion

Table VI presents cancers in relation to smoking and chewing habits in the various religious groups in Bombay. The risk of developing oral and pharyngeal cancers is closely associated with tobacco chewing (Jussawalla & Deshpande, 1971; Wahi, 1968). The low incidence of oral and pharyngeal cancers in Christians and Parsis is perhaps related to the fact that they are less addicted to chewing and known to maintain better oral hygiene (Table VI). In both sexes, cancers of the tongue, buccal mucosa, oropharynx and hypopharynx are commonest in Moslems followed in descending order by the Hindus, Christians and Parsis (Table V). The higher incidence of buccal cancers in the Moslems and Hindus is probably associated with the habit of chewing the betel quid with tobacco and lime and retaining the cud in the buccal sulcus for a long time (Khanolkar, 1950). Such chewing causes attrition of the gums leading to malocclusion of the teeth, which then become sharp-edged. Changes are also seen in the periodontal membranes and the underlying bone. All this ultimately leads to tilting of the teeth either towards the buccal mucosa or the tongue and their sharp edges cause excoriation and ulceration of the adjacent mucosa. Such a traumatic ulcer is then constantly irritated by the chemical products liberated from the betel chew and together with bad oral hygiene, give rise to cancer.

In Christians of both sexes the stomach is the most common site to be involved in the digestive tract. This situation is also observed in many western populations. This viscus is much less affected in the Parsis in whom in both sexes the colon and rectum are commonly affected as a majority of this group is non-vegetarian. In Bombay, pan chewing seems to be strongly indicted as a causative factor in oesophageal cancer (Jussawalla, 1973). The high incidence of cancer at this site in the Hindus and Moslems is noteworthy. Paymaster *et al.* (1968) found from Hospital statistics that Moslems have a high frequency rate of cancer of the oesophagus. The low incidence of oesophageal cancer in the Christians and Parsis is perhaps partly due to lack of addiction to chewing (Table VI).

Cancer of the lung is most commonly seen in Moslem males and Christian females and is the least common in the Parsis of both sexes. A direct relationship between smoking and lung cancer has been demonstrated by numerous epidemiologists.

**Table VI** Cancers in relation to smoking and chewing habits, by religion, Greater Bombay, 1973–78

| *Primary site* | *Religion* | *Chewers only* | | *Chewers and smokers* | | *Smokers only* | | *No habit* | | *Total cancer cases* |
|---|---|---|---|---|---|---|---|---|---|---|
| Tongue | Christian | 2 | (4.9) | 5 | (12.2) | 30 | (73.2) | 4 | (9.7) | 41 |
| | Moslem | 15 | (11.4) | 41 | (31.0) | 67 | (50.8) | 9 | (6.8) | 132 |
| | Hindu | 98 | (19.7) | 111 | (22.3) | 247 | (49.7) | 41 | (8.3) | 497 |
| | Other | 10 | (18.2) | 12 | (21.8) | 24 | (43.6) | 9 | (16.4) | 55 |
| | Total | 125 | (17.2) | 169 | (23.3) | 368 | (50.8) | 63 | (8.7) | 725 |
| Buccal Mucosa | Christian | 1 | (4.5) | 2 | (9.1) | 17 | (77.3) | 2 | (9.1) | 22 |
| | Moslem | 11 | (18.3) | 18 | (30.0) | 28 | (46.7) | 3 | (5.0) | 60 |
| | Hindu | 97 | (37.0) | 69 | (26.3) | 77 | (29.4) | 19 | (7.3) | 262 |
| | Other | 7 | (31.8) | 5 | (22.7) | 9 | (40.9) | 1 | (4.5) | 22 |
| | Total | 116 | (31.7) | 94 | (25.7) | 131 | (35.8) | 25 | (6.8) | 366 |
| Oropharynx | Christian | — | — | 2 | (14.3) | 12 | (85.7) | — | — | 14 |
| | Moslem | 15 | (16.3) | 29 | (31.5) | 45 | (48.9) | 3 | (3.5) | 92 |
| | Hindu | 33 | (14.5) | 59 | (26.0) | 118 | (52.0) | 17 | (7.5) | 227 |
| | Other | 6 | (21.4) | 7 | (25.0) | 13 | (46.4) | 2 | (7.2) | 28 |
| | Total | 54 | (15.0) | 97 | (26.9) | 188 | (52.0) | 22 | (6.1) | 361 |
| Hypopharynx | Christian | 1 | (3.1) | — | — | 28 | (87.5) | 3 | (9.4) | 32 |
| | Moslem | 15 | (10.1) | 52 | (35.1) | 62 | (41.9) | 19 | (12.9) | 148 |
| | Hindu | 126 | (26.0) | 102 | (21.1) | 208 | (43.0) | 48 | (9.9) | 484 |
| | Other | 21 | (29.2) | 16 | (22.2) | 25 | (34.7) | 10 | (13.9) | 72 |
| | Total | 163 | (22.1) | 170 | (23.1) | 323 | (43.9) | 80 | (10.9) | 736 |
| Oesophagus | Christian | — | — | — | — | 52 | (96.3) | 2 | (3.7) | 54 |
| | Moslem | 23 | (21.5) | 27 | (25.2) | 44 | (41.1) | 13 | (12.2) | 107 |
| | Hindu | 116 | (21.0) | 101 | (18.2) | 245 | (44.2) | 92 | (16.6) | 554 |
| | Other | 7 | (12.7) | 12 | (21.8) | 18 | (32.7) | 18 | (32.7) | 55 |
| | Total | 146 | (19.0) | 140 | (18.2) | 359 | (46.6) | 125 | (16.2) | 770 |
| Larynx | Christian | 1 | (3.3) | 3 | (10.0) | 23 | (76.7) | 3 | (10.0) | 30 |
| | Moslem | 20 | (15.8) | 37 | (29.1) | 60 | (47.2) | 10 | (7.9) | 127 |
| | Hindu | 98 | (22.3) | 87 | (19.8) | 203 | (46.3) | 51 | (11.6) | 439 |
| | Other | 11 | (22.0) | 9 | (18.0) | 17 | (34.0) | 13 | (26.0) | 50 |
| | Total | 130 | (20.1) | 136 | (21.1) | 303 | (46.9) | 77 | (11.9) | 646 |
| Lung | Christian | 1 | (1.5) | 2 | (3.0) | 56 | (83.6) | 8 | (11.9) | 67 |
| | Moslem | 6 | (4.6) | 25 | (19.1) | 92 | (70.2) | 8 | (6.1) | 131 |
| | Hindu | 46 | (11.3) | 54 | (13.3) | 259 | (63.6) | 48 | (11.8) | 407 |
| | Other | — | — | 3 | (8.3) | 27 | (75.0) | 6 | (16.7) | 36 |
| | Total | 53 | (8.3) | 84 | (13.1) | 434 | (67.7) | 70 | (10.9) | 641 |
| All related sites | Christian | 6 | (2.3) | 14 | (5.4) | 218 | (83.8) | 22 | (8.5) | 260 |
| | Moslem | 105 | (13.2) | 229 | (28.7) | 398 | (49.9) | 65 | (8.2) | 797 |
| | Hindu | 614 | (21.4) | 579 | (20.2) | 1357 | (47.3) | 316 | (11.1) | 2866 |
| | Other | 62 | (19.5) | 64 | (20.1) | 133 | (41.8) | 59 | (18.6) | 318 |
| | Total | 787 | (18.6) | 886 | (20.9) | 2106 | (49.7) | 462 | (10.8) | 4241 |

Percentages in parenthesis.

Recently, Notani & Sanghvi (1974) and Jussawalla & Jain (1979) have also shown that the risk from lung cancer is maximal amongst smokers in the Bombay area. In both sexes, cancer of the larynx is commonest in Moslems and is seen in decreasing order in the Hindus, Christians and Parsis. It seems that the risk of developing laryngeal cancer is greater if the pan chewer is also addicted to smoking (Table VI). It does not seem to matter if the pan quid is chewed with or without tobacco, the risk being maximal in those addicted to both these habits (Jussawalla & Deshpande, 1971).

**Table VII** Comparison of demographic characteristics related to cervical and breast cancers by religion, Greater Bombay, 1973–1978

| *Demographic characteristic* | *Cervix* | | | | *Breast* | | | |
|---|---|---|---|---|---|---|---|---|
| | *Christian* | *Moslem* | *Hindu* | *Parsi* | *Christian* | *Moslem* | *Hindu* | *Parsi* |
| Proportion of unmarried women to the total population | 5.80 | 0.00 | 0.60 | 16.60 | 6.30 | 5.00 | 5.10 | 18.90 |
| Average age at marriage | 18.70 | 15.11 | 14.68 | 21.30 | 22.31 | 17.56 | 15.11 | 24.10 |
| Average age at first pregnancy | 21.20 | 18.80 | 18.09 | 23.50 | 24.91 | 20.79 | 20.20 | 25.71 |
| Average age at last pregnancy | 32.82 | 30.36 | 29.56 | 34.50 | 31.31 | 30.03 | 29.18 | 32.50 |
| Average number of pregnancies | 4.90 | 5.40 | 5.89 | 4.62 | 4.10 | 4.62 | 5.24 | 4.08 |
| Average spacing of pregnancies (years) | 3.42 | 3.40 | 3.10 | 4.08 | 3.80 | 3.52 | 3.20 | 4.12 |

In Table VII a comparison is made of demographic characterstics of cervical and breast cancer patients in various religious groups in Greater Bombay.

The highest incidence of breast cancer is seen in Parsi women followed by the Christian, Moslem and Hindu females in descending order. Paymaster & Sanghvi (1964) found from hospital statistics that almost one half of all cancers in Parsi women occur in the breast, whilst the cervix is affected in only 17%. This proportion is reversed in Hindu women in whom the cervix is the seat of cancer in 50% and the breast in 14%. It has been repeatedly observed that the incidence of breast cancer is higher in unmarried women. Our findings also concur with the view that women with breast cancer have low parity and that higher age at first pregnancy is an important risk factor (Table VII). The estimated risk for women who deliver for the first time at the age of 30 or later is nearly double that of those who bear their first child before the age of 20 (Jussawalla *et al.*, 1980*a*, Jussawalla & Yeole, 1984). More women seem to remain unmarried in the Parsi and Christian communities than in the Moslem and Hindu groups. The average age at marriage is also higher among the Parsi and Christian women and over the years increasingly larger numbers of women seem to marry late. These social factors perhaps have a bearing on the high incidence of cancer of the breast in Christian and Parsi women.

It is common experience that cancer of the cervix is predominantly a disease of married women, especially occurring in those who marry at an early age and bear a large number of children (Jussawalla *et al.*, 1971). Whatever influence marital status, active sexual life and child bearing may have on the occurrence of cervical cancer, the suspect factors apparently begin to exert their action at a much earlier age. The low risk in Parsi and Christian women as compared with the Hindu are probably due to their higher age at marriage and at first pregnancy, broader spacing of pregnancies, fewer number of pregnancies and perhaps better hygiene and care during delivery (Table VII).

The Moslem rates at the sites involving the male genital organs (grouped together) are lower than the figures reported for the other religious groups. In all the groups taken together, cancer of the prostate is predominant. Penile cancer, however, is almost non-existent in Moslems mostly due to the practice of circumcision. It is also rare in the Parsis, perhaps due to better hygiene practised by a more affluent and literate group.

The higher incidence of bladder cancer in Christians may perhaps be attributed to their almost universal smoking habit. The incidence of skin cancer is higher in the Parsis and lower in the Christians, as Parsis have a relatively fairer complexion. The higher incidence of this cancer in Parsis who have less cutaneous pigmentation is due to the accepted fact that carcinogenic action of the ultraviolet content of sunlight, in the relative absence of skin pigmentation in a tropical country such as India, leads to a relatively higher incidence of skin cancer.

**References**

JUSSAWALLA, D.J., HAENSZEL, W., DESHPANDE, V.A. & NATEKAR, M.V. (1968). Cancer incidence in Greater Bombay: Assessment of cancer risk by age. *Br. J. Cancer*, **22**, 623.

JUSSAWALLA, D.J., DESHPANDE, V.A., HAENSZEL, W. & NATEKAR, M.V. (1970). Differences observed in the site incidence of cancer between Parsi community and the total population of Greater Bombay: A critical appraisal. *Br. J. Cancer*, **24**, 56.

JUSSAWALLA, D.J. & DESHPANDE, V.A. (1971). Evaluation of cancer risk in tobacco chewers and smokers: An epidemiologic assessment, *Cancer*, **28**, 244.

JUSSAWALLA, D.J. (1973). Epidemiological assessment of the etiology of oesophageal cancer in Greater Bombay in *Monograph on Epidemiology of Oesophageal Cancer*, Indian Cancer Society, Bombay.

JUSSAWALLA, D.J. & JAIN, D.K. (1977). Breast cancer and religion in Greater Bombay women – An epidemiological study of 2130 women over a 9 year period. *Br. J. Cancer*, **36**, 634.

JUSSAWALLA, D.J. & JAIN, D.K. (1979). Lung cancer in Greater Bombay: Correlations with religion and smoking. *Br. J. Cancer*, **40**, 437.

JUSSAWALLA, D.J., YEOLE, B.B., NATEKAR, M.V. & RAJAGOPALAN, T.R. (1980*a*). Differences in site patterns of cancer in Sindhi and Parsi sub-groups and the general population of Greater Bombay. *Indian J. Cancer*, **17**, 78.

JUSSAWALLA, D.J., YEOLE, B.B., NATEKAR, M.V. & RAJAGOPALAN, T.R. (1980*b*). Cancer in the Sindhi population of Greater Bombay. *Cancer*, **46**, 2107.

JUSSAWALLA, D.J. & YEOLE, B.B. (1984). Epidemiology of cancer of the cervix in Greater Bombay. *J. Surg. Oncol.*, **26**, 53.

JUSSAWALLA, D.J., YEOLE, B.B. & NATEKAR, M.V. (1985). Cancer in Indian Moslems. *Cancer*. **55**, 1149.

KHANOLKAR, V.R. (1950). Cancer in India, *Acta Un Internat. Ctr. Cancerum*, **6**, 881.

NOTANI, P. & SANGHVI, L.D. (1974). A retrospective study of lung cancer in Bombay. *Br. J. Cancer*, **29**, 477.

PAYMASTER, J.C. & SANGHVI, L.D. (1964). Cancer and its distribution in India. *Cancer*, **17**, 1026.

PAYMASTER, J.C. SANGHVI, L.D. & GANGADHARAN, P. (1968). Cancer in the gastrointestinal tract in Western India, *Cancer*, **21**, 279.

RELE, J.R. & KANITKAR, T. (1980). *Fertility and Family Planning in Greater Bombay*. Popular Prakashan Private Limited, 44.

WAHI, P.N. (1968). The epidemiology of oral and oropharyngeal cancer: A report of the study in Mainpuri District, Uttar Pradesh, India. *Bull WHO*, **38**, 495.

WATERHOUSE, J., MUIR, C., CORREA, P. & POWELL, J. (1976). *Cancer Incidence in Five Continents*, Vol. III, IARC, Lyon.